# Complete mitochondrial genome of the freshwater bryozoan *Pectinatella magnifica* (Phylactolaemata: Plumatellida) assembled from next-generation sequencing data

**DOI:** 10.1080/23802359.2018.1450657

**Published:** 2018-03-15

**Authors:** Jeong-Soo Gim, Eui-Jeong Ko, Hyo-Gyeum Kim, Young-Min Kim, Sungwon Hong, Hyun-Woo Kim, Jeong-An Gim, Gea-Jae Joo, Hyunbin Jo

**Affiliations:** a Department of Integrated Biological Science, Pusan National University, Busan, Republic of Korea;; b Department of Environmental Education, Sunchon National University, Suncheon, Republic of Korea;; c Graduate School of Convergence Science and Technology, Seoul National University, Suwon, Republic of Korea;; d Department of Bioscience, Aarhus University, Silkeborg, Denmark

**Keywords:** Complete mitochondrial genome, *Pectinatella magnifica*

## Abstract

The complete mitochondrial genome of the freshwater bryozoan *Pectinatella magnifica* was sequenced. The circular mitochondrial genome is 17,539 bp and consists of 13 protein-coding, two ribosomal RNA, and 22 transfer RNA genes (GenBank accession no. MG546680). The Bayesian comparative analysis of molecular evolution rates revealed no acceleration of the mitochondrial DNA (mtDNA) evolution of *P. magnifica*. Results of maximum likelihood analysis showed that this species clustered with other species of the phylum Bryozoa.

The freshwater bryozoan *Pectinatella magnifica* (Leidy 1851) is a member of the phylum Bryozoa. Molecular barcoding approach in the mitochondrial region was recently introduced to discriminate between bryozoan species (Fuchs et al. 2009; Lee et al. 2011; Waeschenbach et al. 2012). Phylogenetic relationships in the phylum Bryozoa, however, are still poorly understood except for the cytochrome c oxidase subunit I (COI) gene (Kang and An 2015). Furthermore, complete sequences of mitochondrial genomes (mitochondrial DNA (mtDNA)) of *P. magnifica* were not determined. In this study, we described a new complete mtDNA of *P. magnifica*, assembled using next-generation sequencing. This result will be valuable for further phylogenetic analyses of Bryozoa.

*Pectinatella magnifica* is one of the most abundant freshwater bryozoan species in South Korea and is distributed in lotic and lentic habitats on natural and artificial submerged surfaces (Kang and An 2015). A colony of *P. magnifica* specimen was collected from the Miryang River, South Korea (35°28′14.35′′N, 128°45′43.24′′E) in August 2016. The collected specimen was kept in a 45 × 30 cm sized tank in a temperature-controlled room (16.4 ± 1 °C) under a natural photoperiod and aeration with a one-day acclimatization period. Then, we collected the larvae of the *P. magnifica* specimen and placed them in individual tanks. Total DNA was extracted from the *P. magnifica* larvae using modified 95% ethyl alcohol and was stored in Specimen Museum of Pusan National University (accession number: PNUBIO-0110010101). The library preparation and DNA sequencing (100 bp mate pairs with different insert sizes, Illumina HiSeq4000, San Diego, CA) were performed at Macrogen Inc. (Seoul, Korea).

The genome was assembled using the MEGA6 software (Tamura et al. 2013). The phylogenetic tree was analysed using 13 protein-coding gene sequences using maximum likelihood (Kimura 1980). The annotated mitochondrial genome sequence of *P. magnifica* is available at the National Center for Biotechnology Information (NCBI) database (GenBank accession number MG546680). The mtDNA of *P. magnifica* is 17,539 bp and contains two rRNA, 22 tRNA, and 13 protein-coding genes. The total length of the intergenic regions is 3367 bp, which constitutes 19.20% of the genome. The A + T base composition of the genome is 62.88%, the A + T content of genes ranges from 55.38% to 75.38%. The most common start codon was ATG. However, four genes were AAT, ATA, and ATT. Two genes were terminated with a complete TGA stop codon, and the rest were incomplete. The total base pair of the tRNA was comparable to that of the closely related mitochondrial genome of *Bugula neritina*. However, the trnY was 20 bp larger (Jang and Hwang 2009). *Pectinatella magnifica* is not clustered on the phylogenetic tree with other species of the phylum Bryozoa (Figure 1). Considering that the genome of the family of *P. magnifica* has been poorly understood, the elucidation of the complete mitochondrial genome sequence of this species will be extremely useful for future Bryozoa phylogeographical studies.

**Figure 1.**
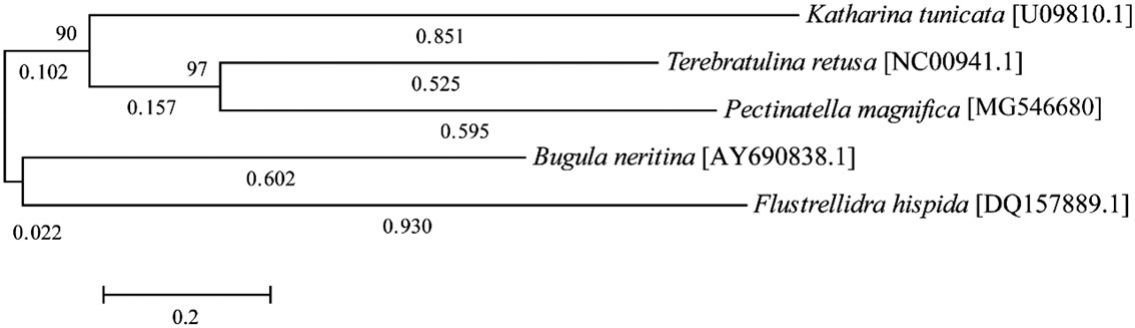
Maximum likelihood trees inferred from coding sequences of all 13 mitochondrial genes of five species of Bryozoa; *Terebratulina retusa* (NC00941.1), *Katharina tunicata* (U09810.1), *Bugula neritina* (AY690838.1), and *Flustrellidra hispida* (DQ157889.1) as the out group. The heuristic search (using the 50% majority-rule with 1000 bootstrap replicates) shows *P. magnifica* (MG546680) as a related species to the phylum Bryozoa, with a relationship similar to that of other phylogenies.
